# Feasibility of peer support services among people with severe mental illness in China

**DOI:** 10.1186/s12888-019-2334-x

**Published:** 2019-11-14

**Authors:** Yunge Fan, Ning Ma, Liang Ma, Wufang Zhang, Wei Xu, Ruina Shi, Hanyan Chen, J. Steven Lamberti, Eric D. Caine

**Affiliations:** 1 Peking University Sixth Hospital, Peking University Institute of Mental Health, Key Laboratory of Mental Health, Ministry of Health (Peking University), Haidian District Huayuanbeilu No.51, Beijing, 100191 China; 2 Division of Psychology, Nanyang Technological University, Singapore, Singapore; 3 Third hospital of Chaoyang District, Beijing Chaoyang District Mental Disease Prevention and Control Center, Beijing, China; 4 Puyang Mental Health Center, Puyang, Henan China; 5 Beihai Hepu Psychiatric Hospital, Beihai, Guangxi China; 6 Department of Psychiatry, University of Rochester Medical Center, New York, USA

**Keywords:** Peer support service, Community, Severe mental illness, Effectiveness, China

## Abstract

**Background:**

Peer-delivered services potentially provide broad, multifaceted benefits for persons suffering severe mental illness. Most studies to date have been conducted in countries with well-developed outpatient mental health systems. The objective of this study was to examine the feasibility for developing a community-based peer service in China.

**Methods:**

Thirteen peer service providers and 54 consumers were recruited from four communities in Beijing. We initiated the program in two communities, followed by another two in order to verify and add to our understanding of potential scalable feasibility. Semi-structured face-to-face interviews were conducted 12 month after initiation at each site to measure satisfaction and perceived benefits from perspectives of peer service providers, and consumers and their caregivers.

**Results:**

Key stakeholders reported that peer support services were satisfying and beneficial. Eleven of 13 peer service providers were willing to continue in their roles. Ten, 8, and 7 of them perceived improvements in working skills, social communication skills, and mood, respectively. Among consumers, 39 of 54 were satisfied with peer services. Improvements in mood, social communication skills, illness knowledge, and illness stability were detected among 23, 18, 13, and 13 consumers, respectively. For caregivers, 31 of 32 expressed a positive view regarding peer services. Caregivers reported improvement in their own mood, confidence in recovery of their family members, and reduction in caretaker burdens.

**Conclusions:**

The findings highlight that peer-delivered services have promise in China for benefiting persons with severe mental illness and their family caregivers, as well as the peer service providers themselves.

## Background

Peer support services delivered to persons with severe mental illness (SMI) are based on the premise that individuals who have overcome or recovered from the effects of a mental illness can offer useful support, encouragement, and mentoring to others similarly affected [1]. Peer services have been included as key components of supportive networks and recovery-oriented services for individuals with SMI in many Westernized countries that have well-developed ambulatory mental health systems [2–4]. Peer service providers serve as positive role models by improving confidence for recovery, thus providing hope, together with reducing perceived stigma [5].

We anticipated in our study that three groups might benefit from the development of services-peer service providers themselves, recipients of support (hereafter, “consumers”), and their family caregivers. Prior work has shown that peer service providers have felt more empowered, autonomous, and competent/confident, resulting in higher self-esteem from helping others [6, 7]. They have become more confident in their own recovery, solidifying daily life skills and learning to focus more on their own and others’ strengths [5]. Notably, they also have been observed to have a high rate of turnover. This may reflect a reduction in psychiatric symptoms and increase in social functioning, as many move to other higher salaried jobs [8]. But it also may indicate dissatisfaction or a misfit between job requirements and personal needs. We studied this issue as one aspect of our study.

Consumers of peer support services have reported a greater sense of feeling understood, respected, and trusted [9–11]. One study revealed statistically significant improvements for participants in self-esteem, self-efficacy, social support, and spiritual well-being [10]. Another, however, showed no statistically significant differences on social functioning among participants suffering SMI, even as they reported improved self-efficacy [11].

Families of people with SMI experience substantial caregiver burden [12, 13]. They report both emotional distress [14] and poor perceived quality of life [15]. Of note, an Australian peer support program that interviewed family caregivers found high satisfaction with the quality of service and reported that the service gave hope to them and to their mentally ill family members. Information and support provided by peer service providers was perceived to be invaluable [5].

Nevertheless, there are challenges evident when considering the generalizability of previous studies of peer-delivered services. The majority have come from Western countries with well-developed ambulatory services; involvement of peers in care is new to most nations that now are developing outpatient programming, and may not conform to cultural notions of family privacy or hierarchical approaches to medical and psychiatric care systems. Prior research has indicated that the operation of peer services should be consistent with local customs, values, and resource availability [16]. Attempts to implement peer services were made in Hong Kong; these focused primarily on changing attitudes among peer support workers toward the delivery of peer services [17]. However, there was no clarity regarding their acceptability or the overall impact of peer services delivered in Chinese communities. Inconsistent outcomes also are reported for participants of previous studies on the effectiveness of peer services [18]: Some demonstrated improvements in social functioning and quality of life [10], while others have not [11]. And few studies have examined the effect of peer support service on caregivers, especially the specific impact on their emotions or perceived quality of life apart from their attitudes or satisfaction with services.

The primary objective of this project, based in Beijing, involved demonstrating the feasibility of community-based peer support services among persons with SMI. We assessed feasibility by measuring of satisfaction and perceived benefit while also assessing elements necessary to develop and sustain the project. The initial implementation of our model of peer support has been described previously [19].

## Methods

### Participants

**Fig. 1:**
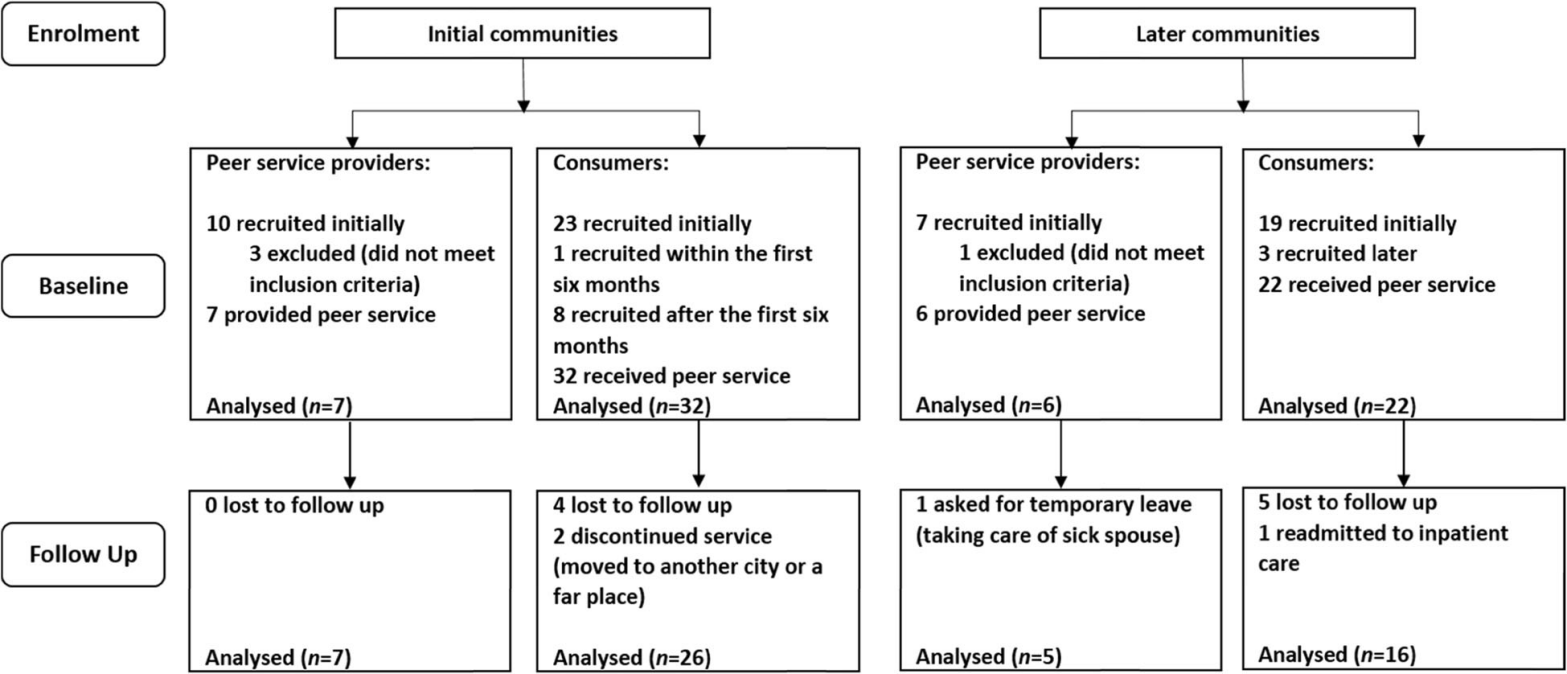
Enrolment and flow of participants

Participants were patients with SMI recruited from communities in Chaoyang District, Beijing. As one of the core areas and the most populous district in Beijing, Chaoyang District consists of 43 communities with the population of 3.74 million [20]. Peer support services were initiated in two communities (Tuanjiehu and Maizidian) (initial communities) in July 2013. Seven peer service providers (five from Tuanjiehu and two from Maizidian) and 32 consumers (patient-recipients) were recruited from the two communities. Considering the relatively small samples and the target of verifying and supplementing the outcomes of the peer support service, peer support services were initiated in another two communities (Xiangheyuan and Jingsong) in August 2015 (later communities), and six peer service providers (four from Xiangheyuan and two from Jingsong) and 22 consumers were recruited. Therefore, a total of 13 peer service providers and 54 consumers were recruited. In each community in Chaoyang District, one or two community doctors are assigned to provide follow-up services for the patients with SMI; and there is one community activity rehabilitation center in each community; the four communities did not differ prominently in mental health resources. All peer service providers and consumers still received usual care throughout the entire study. The enrolment process for participants is shown in Fig. 1.

All peer service providers were recommended by community doctors. In addition to recommendation from community doctors, posters were placed in diverse locations in each community to recruit consumers. Qualifications of peer service providers include: diagnosed with schizophrenia or bipolar disorder according to the record provided by community doctors; age between 18 and 60 years old; stable at least 6 months; being adherent to medications according to patients’ and family members’ reports; having insight about their illnesses, which was assessed via individual interviews conducted by a psychiatrist in the research team; no drug or alcohol abuse; no severe physical illness; and having good social functioning, which was defined as a score of over 50 on the personal and social performance scale (PSP) [21]. All participating peer service providers were expected to be compassionate and willing to help others. Applicants with strong practical skills (e.g., cooking, drawing) were preferred. The inclusion criteria of consumers included: diagnosed with schizophrenia or bipolar disorder; age 18–60 years old; stable at least 3 months; no drug or alcohol abuse; and no severe physical illness. Caregivers (parents or spouse) of providers and consumers were also recruited if they were willing to share their views in the follow-up procedure.

The project proposal was reviewed and approved by the Ethics Committee of Peking University Sixth Hospital prior to initiation. All peer service providers and consumers provided written informed consent, their caregivers also provided written or verbal informed consent.

### Peer support service intervention

A total of 214 peer support services were delivered. Most (98.6%) of the services were conducted in group. Each service session typically involved at least two service providers, one of whom leads and the other assists in service delivery, record keeping, and documenting of consumers’ feedback. The ratio of peer service providers to consumers was between 1:3 and 1:5 in each community. The peer services were delivered once every one or 2 weeks, and participants’ attendance rate was required to be more than 40%. The majority of services were held in community rehabilitation centers or community health care centers, except for outdoor activities. Before providing each service, peer service providers should complete a Service Plan Form and submit it to their community doctors, and then the community doctors or social workers would instruct peer service providers to prepare for the service. The community doctors should offer non-scheduled supervision depending on peer service providers’ needs.

The two initial communities and two later communities differed in service schedules and targeted contents in the early stages. For the initial communities, the service schedules or the targeted contents were not limited at first, to allow providers’ substantial latitude in their roles, thus resulting in a slow ramp-up of services especially during the first 6 months. Therefore, after such a period of time and when peer support services were implemented in the later communities, a schedule with a fixed service timetable (once every one or 2 weeks) was suggested, to provide the service providers with eight choices of service contents, including: daily life skills, social skills, knowledge of mental disorders, entertainment, fine motor skill practice, personal perceptions, healthy life style support, and emotional support. Providers were expected to select them according to their own interest or strength. All the eight options were delivered, and the top three most popular categories were fine motor skill practice and exercise (19%), daily life skills study (16%), and emotional support (15%). The format of each activity varied depending on the topics, including group discussion, role play, personal sharing, lecture, and outdoor exercises. The duration of each service ranged from 40 min to 120 min. Detailed information regarding the peer service program was previously reported in another paper [19].

### Procedure

After recruitment, all peer service providers and consumers were evaluated by a psychiatrist in the research team, to determine their eligibility for participating in the study. All peer service providers were then required to attend an intensive pre-service training offered in five half-days by a psychiatrist and a clinical psychologist. Training contents included the concept and theory of peer support services, working principles and requirements (including confidentiality, boundaries, and relationships), how to design and implement group activities, effective listening and speaking skills, how to answer patients’ questions about mental health, how to handle emergency situations, and how to complete the record forms. After satisfactory completion of the training, they were eligible to provide peer support interventions. There were no candidates for peer service providers who were excluded during the training period, and as previously reported in greater details, peer service providers were supervised throughout the delivery process [19].

Peer service providers, consumers, and their caregivers from the two initial communities were planned to be evaluated at 12 months. However, their evaluation was postponed to 18 months due to the slow ramp-up of services during the first 6 months and inexperience of the staff. During the same period of time, peer service providers and consumers, and their caregivers were also recruited from the later communities, and were ultimately evaluated during the same block of time, 18 months after inception of participants from the initial communities and 12 months for the later two communities.

### Outcome measures

All the evaluations involved semi-structured face-to-face interviews by psychiatrists and psychologists in the research team, which were conducted at a time when peer services were on-going. The service satisfaction and perceived benefits were measured from the perspectives of peer service providers, consumers, and their caregivers. The measures were primarily developed based on prior studies, which measured service satisfaction and perceived benefits through structured interviews [5, 22, 23]. Then, the researchers specializing in rehabilitation of psychiatric disorders and qualitative studies, and the staffs involving in the project (e.g., community doctors, project coordinator, etc.) were invited to go through and revise the items, to make sure they were understandable in Chinese context. Finally, the items were discussed with several peer service providers to determine whether the questions were answerable.

#### Service satisfaction

##### Peer service providers

Service satisfaction among peer service providers was measured in three self-reported areas: overall work satisfaction, work competency and stress, and willingness to continue working as a peer service provider. Overall work satisfaction was assessed by one “yes/no” question (“Are you satisfied with the provided peer service?”), and another six questions, to evaluate specific areas of service satisfaction including time, content, environment, relationship with consumers, relationship with other peer service providers, and relationship with doctors involved in the service. Each specific question was scored from 1 (extremely unsatisfied) to 5 (extremely satisfied). For work competency and stress, two “yes/no” questions were asked, with a follow-up question depending on answers: “Do you think you are qualified to be a peer service provider?” “Is it stressful to be a peer service provider? (if yes) Please describe the stress or difficulty.” Willingness to continue working as a peer service provider was evaluated by two questions: “Are you willing to continue being a peer service provider and delivering peer support service? (yes-or-no)” and “From 0 to 10, how confident do you feel that you can continue helping other consumers?”

##### Consumers

Their service satisfaction was measured through self-report in two areas: overall service satisfaction and willingness to continue the participation in the program. Overall service satisfaction was assessed by one “yes/no” question (“Are you satisfied with the peer service providers and peer service they delivered?”), and another five questions, to evaluate the specific service satisfaction including peer service providers’ competence, speaking manners, disease stability, service punctuality, and richness of activity topic. Each specific question was scored from 1 (extremely unsatisfied) to 5 (extremely satisfied). The willingness to continue the participation in the program was assessed by one “yes/no” question (“Are you willing to continue participating in peer support service delivered by the peer service providers?”).

##### Caregivers

(If available) Caregivers of peer service providers and consumers were evaluated. A “yes/no” question was asked to assess their attitudes towards peer services, followed by different questions for families of peer service providers or consumers: “Are you willing to ask your family member to continue participating in peer support service delivered by the peer service providers?” For caregivers of peer service providers: “In your opinion, is your family member qualified to be a peer service provider?” For caregivers of consumers: “Do you expect your family member to become a peer service provider and deliver peer support services in the future?”

#### Perceived benefits

##### Peer service providers

The interview for peer service providers consists of two parts. Firstly, measure their self-perceived benefit through four areas: disease related benefit, social communication, daily life and work functioning, emotion and self-perception. For disease related benefit, two questions were asked: Could the participating in the service help you gain more knowledge about mental illness, and help your illness become more stable? For social communication, three questions were asked: Could the participating in the service improve your social communication skills, improve relationships with your family members, and promote your caring for and supporting of others? For daily life and work functioning, two questions were asked: Could the participating in the service improve your independent living skills, and improve your working skills? For emotion and self-perception, three questions were asked: Could the participating in the service make you more confident and positive about the recovery process, improve your mood and feelings that you are cared for by others, and improve your sense of belonging and social inclusion? Service providers were expected to answer yes-or-no for each question and to give reasons or examples, and evaluators recorded their responses verbatim.

The second part was to assess service providers’ understanding about perceived benefit from others’ perspectives. For example, the following questions should be asked: “Did your family members/friends/community doctors/other community staff think you have any improvement after participating in peer services? What did they appraise?” Evaluators recorded verbatim their words and sentences.

##### Consumers

The measures of perceived benefits for consumers were identical with those for peer service providers.

##### Caregivers

The interview for caregivers also consists of two parts. The first part was identical with that for peer service providers. All caregivers were asked to share their opinions about the effects of peer services on their participating family members.

The second part was to assess the effects of peer support service on themselves. All caregivers were asked to answer the following six questions: Could your family members’ participation in the service help you gain more knowledge about mental illness, improve the relationship between you and your affected family member, and make your affected family member more independent, thus reducing your burden and time devoted to providing care? Did it improve your quality of daily life, improve your own mood, and make you more confident and positive about the recovery process of your affected family member? Caregivers were asked to answer “yes-or-no” for each question, and provide reasons or examples. Evaluators recorded verbatim their words and sentences.

### Statistical analyses

Continuous variables were reported as means ± SD, discrete variables were reported as N’s and percentages. Chi-square test and Mann Whitney U tests or independent *t* tests were performed to detect differences between participants who continued and those who dropped out before follow-up evaluations. The same analyses were conducted to examine whether the results of initial communities could be verified by the later ones. The differences between peer service providers and consumers in perceived benefits were also examined. Correction for multiple testing was achieved using the Bonferroni.

## Results

### Participant characteristics and response rates

The descriptive statistics for key variables of participant characteristics are listed in Table 1. The detailed information for participants from the initial and later communities are listed in Additional file 1: Table S1. There was no significant difference in all demographic variables between those from the initial and later communities for both peer service providers and consumers (Bonferroni-corrected threshold of *p* < 8.33 × 10^− 3^). Seven peer service providers (100%) from the initial communities continuously provided peer support services and completed the 18-month evaluation, while one peer service provider from the later communities asked for a temporary leave due to taking care of sick spouse, resulting in five peer service providers (83%) completing the follow-up evaluation at 12 months. Among consumers from the initial communities, 26 of 32 (81%) completed the follow-up evaluations. Among the later group, 16 of 22 (73%) consumers completed the follow-up evaluations at 12 months. Response rates showed no significant difference between the two groups among both peer service providers (*χ*^2^(1) = 1.264, *p* = 0.462) and consumers (*χ*^2^(1) = 0.548, *p* = 0.517). There also were no significant differences in any of the baseline demographic variables between those consumers who continued and those dropouts at follow-up evaluations for participants from the initial (*ps* > 0.078) and later communities (*ps* > 0.173).

**Table 1:** Descriptive statistics of the participants’ demographic at baseline

Demographic variables	Peer service provider (*n* = 13)		Consumer (*n* = 54)	
	N (%)	Mean (SD)	N (%)	Mean (SD)
Age		38.92 (9.61)		46.59 (8.27)
Gender				
Female	5 (38.5%)		31 (57.4%)	
Male	8 (61.5%)		23 (42.6%)	
Education				
≤ Middle school	2 (15.4%)		13 (24.1%)	
High school	8 (61.5%)		25 (46.3%)	
Some college	1 (7.7%)		8 (14.8%)	
College or above	2 (15.4%)		8 (14.8%)	
Current marital status				
Never married	8 (61.5%)		28 (51.9%)	
Currently married	4 (30.8%)		14 (25.9%)	
Divorced or separated	1 (7.7%)		12 (22.2%)	
Lives alone	0 (0%)		10 (18.5%)	
Current employment				
Yes	4 (30.8%)		5 (9.3%)	
No	9 (69.2%)		49 (90.7%)	
Diagnosis				
Schizophrenia	8 (61.5%)		48 (88.9%)	
Bipolar disorder	5 (38.5%)		6 (11.1%)	
First onset age		20.92 (5.35)		25.15 (8.68)

### Peer service providers

Results of service satisfaction among peer service providers at follow-up evaluations are shown in Table 2. All peer service providers felt satisfied with themselves and the peer services they delivered. In specific, most of average scores were equal to or higher than 4 (of 5), which represents general satisfaction except for the service content which only attained an average value of 3.42. Ten of the 12 service providers (83%) felt that they were qualified for the role, though half of them reported the existence of stress. Regarding the reasons for stress or difficulty functioning as a peer service provider, five of them (42%) thought they had a poor organizing ability, three (25%) reported a lack of confidence, and two (17%) found it was difficult to come up with an activity topic. For willingness to continue working as a peer service provider, 11 of the 12 (92%) wished to continue peer work. One reported that there was too much pressure and showed a desire to quit peer service provider group and join in the consumer group. The self-reported scores regarding self-confidence in continuing working as a provider were relatively high (7.13), especially in the later communities (7.80). There was no significant difference in service satisfaction between providers from the initial and those from the later communities (*ps* > 0.080) (as shown in Additional file 1: Table S2).

**Table 2:** Results of service satisfaction among peer service providers at follow-up evaluation

Service satisfaction	Peer service provider (*n* = 12)	
	N (%)	Mean (SD)
Overall work satisfaction		
Overall, satisfied with the peer service	12 (100%)	
Time		4.08 (0.289)
Content		3.42 (0.996)
Environment		4.08 (0.289)
Relationship with consumers		4.08 (0.289)
Relationship with other peer service providers		4.17 (0.577)
Relationship with doctors involved		4.25 (0.452)
Work competency and stress		
Qualified to be peer service providers	10 (83%)	
Feel stressful to be peer service providers	6 (50%)	
Continuous work willingness		
Willing to continue	11 (92%)	
Confidence		7.13 (2.366)

The results of perceived benefit among peer service providers at follow-up evaluations are shown in Table 3. In total, 10 of 12 peer service providers (83%) perceived improvement in working skills. Eight of the 12 (67%) reported an increase in social communication skills. In addition, seven (58%) peer service providers expressed that organizing peer services greatly improved their mood and feelings that they were cared by others. As shown in Additional file 1: Table S3, although peer service providers from the later communities rated slightly higher than the initial ones, no significant difference was detected between the two groups.

**Table 3:** Results of perceived benefit among peer service providers and consumers at follow-up evaluation ^a^

Perceived benefit	Peer service provider (*n* = 12)	Consumer (*n* = 42)
	N (%)	N (%)
Self-perceived benefit		
Disease related		
Know more disease knowledge	6 (50%)	13 (31%)
Disease become more stable	3 (25%)	13 (31%)
Social communication		
Improve social communication skill	8 (67%)	18 (43%)
Improve relationship with families	3 (25%)	9 (21%)
Care and support more for others	5 (42%)	11 (26%)
Ability of daily life and work		
Improve self-living ability	4 (33%)	10 (24%)
Improve work skill ∆	10 (83%)	9 (21%)
Emotion and self-perception		
More confidence about recovery	6 (50%)	11 (26%)
Improve mood and feeling cared about	7 (58%)	23 (55%)
Improve sense of belonging	4 (33%)	13 (31%)
Perceived benefit from others perspective		
Family members	9 (75%)	19 (45%)
Friends	2 (17%)	10 (24%)
Community doctors	7 (58%)	16 (38%)
Other community staff	4 (33%)	13 (31%)

^a^The numbers showed in this table represent how many participants answered “yes” on each particular question

∆ Peer service provider group and consumer group showed significant difference (*p* < 0.001)

### Consumers

Results of service satisfaction among consumers at follow-up evaluations are presented in Table 4. Thirty-nine of 42 (93%) consumers were satisfied with the peer service providers and peer services in their own communities. Notably, if one were to be more conservative and include all initial consumer-participants, 39 of 54 (72%) expressed satisfaction. Scores of specific program aspects ranged from 3.80 to 4.02 (out of 5) for all except for richness of activity topic (3.66). Thirty-six of 42 (86%) consumers expressed their intention to further participate in peer services. As shown in Additional file 1: Table S4, values of service satisfaction rated by consumers in the later communities were slightly higher than the initial ones, although statistically significant differences were only found in service punctuality (*t* = − 3.348, *p* = 0.002) and richness of activity topic (*t* = − 2.853, *p* = 0.007).

**Table 4:** Results of service satisfaction among consumers at follow-up evaluation

Service satisfaction	Consumer (*n* = 42)	
	N (%)	Mean (SD)
Overall work satisfaction		
Overall, satisfied with the peer service providers and service	39 (93%)	
Peer service providers’ competence		3.80 (0.813)
Peer service providers’ speak manners		3.98 (0.524)
Peer service providers’ disease stability		3.85 (0.573)
Service punctuality		4.02 (0.474)
Richness of activity topic		3.66 (0.855)
Continuous participation willingness	36 (86%)	

For the perceived benefit (as shown in Table 3), 23 of 42 (55%) noted improvement in mood and feelings that they were cared by others, which was also rated the highest in both the initial (10 of 26 (39%)) and later (13 of 16 (81%)) communities. As some consumers mentioned that they felt accepted when participating in the activities because they were all patients, this was a setting that they could trust. Eighteen (43%) consumers reported benefits in social communication skills. One consumer stated that he could not even talk with other consumers at the beginning of the service, while he could communicate easily with healthy people now. In addition, 13 (31%) consumers reported benefit in disease-related aspects, such as the improvement in knowledge about mental illness and illness stability. One consumer pointed out that peer services were beneficial to his sleep. Thirteen (31%) consumers reported an improved sense of belonging. Similar to the results of peer service providers, more consumers from the later communities reported an improvement in all items in contrast to those from the initial ones (as shown in Additional file 1: Table S3). For the understanding about perceived benefits from others’ perspectives, 19 (45%) and 16 (38%) consumers reported a benefit from their family members and community doctors’ perspective, respectively.

When comparing differences in perceived benefits among all peer service providers and consumers (as shown in Table 3), it was evident that the proportion of peer service providers who perceived improvements after participating in peer support service was greater than for consumers in almost each item, apart from disease stability (3 (25%) vs. 13 (31%) respectively). However, only the self-perceived increase in work skill was proved to be significant (*χ*^*2*^(1) = 15.684, *p* < 0.001).

### Caregivers

A total of 32 caregivers from 32 families (10 of peer service providers, 22 of consumers), with 21 (66%) being female and average age being 68.4 years, participated in the follow-up evaluations. Thirty-one caregivers (97%) were willing to ask their family members to continue participating in peer support services in one capacity or the other. Only one caregiver from the initial communities showed a negative attitude toward the service because her consumer family member expressed feelings of inferior compared with peer service providers. Among the 10 caregivers of peer service providers, eight (80%) considered that their family members to be qualified were peer service providers, while 14 of 22 (64%) consumers’ caregivers expressed an expectation that their family members would become peer service providers and deliver peer support to others in the future.

In terms of the perceived benefit appraised from caregivers, 12 of 32 (38%) detected improvements in their families’ social communication skills. Eleven (34%) indicated that their family members became more confident about their recovery. And 11 (34%) reported that their family members’ mental illnesses became more stable. As for the service effect on themselves, 14 (44%) reported that family members participating in the service improved caregivers’ own mood. Furthermore, 11 (34%) caregivers reported that they were more confident about the recovery of their family members, and 10 (31%) thought participating in the service helped their family members more independent, thus further reducing the caring time and caregiver burden. Several caregivers indicated that participants helped more with housework, and others experienced more free time to deal with their own affairs after peer services were begun.

## Discussion

This study examined satisfaction and perceived benefits of a community-based peer support service launched in four Beijing communities among patients with SMI. We designed it to be consistent with Chinese culture, which places care for persons with health challenges inside family and social structures. The results revealed high service satisfaction among peer service providers, consumers, and their caregivers. Almost all of them expressed willingness to continue participating in the services. In addition, the results demonstrated apparent improvements in symptoms of mental illness, social communication in the program and often at home, daily life functioning, emotional expression and self-perception, and for the majority of peer service providers, in the context of their work.

For peer service providers, distinct improvements were noted in particular items, including: working skills, social communication skills, and mood and feelings. These outcomes are consistent with most previous studies. Both pre-service training sessions and the process of providing services substantially increased providers’ job performance competency [24], anticipatory socialization [25], and skills in communicating positive regard, understanding, and acceptance to consumers. These, in turn, motivated consumers’ treatment engagement and adherence [26]. In addition, service providers could increase consumers’ sense of hope, belonging and contentment in various life domains, which further improved peer service providers’ own mood [27].

For consumers, benefits were especially demonstrated in mood and feelings about being cared by others, social communication skills, knowledge of illness and illness stability, and sense of belonging, which were consistent with evidence from prior studies [28, 29]. For illness stability, although previous studies showed inconsistent conclusions about whether peer service could reduce rehospitalization and overall psychiatric symptoms [11, 18, 30, 31], peer services have indeed been proven to be helpful for engaging consumers into healthcare [27], with improved longitudinal treatment motivation and higher service attendance [26]. The current study introduced a new illness related indicator-knowledge of mental illness. This was also tracked during peer services because of strong association between knowledge and treatment adherence [32].

Comparisons between service effects on peer service providers and consumers revealed that the current program was beneficial, but targeted at different areas of knowledge and functioning within each group. Generally speaking, compared with the consumers, peer service providers were more likely to benefit in areas other than disease stability, which probably was due to a ceiling effect that resulted from the peer service providers’ better initial illness stability. Furthermore, working skill was the only area that showed significant differences between peer service providers and consumers, and it was consistent with providers’ work experience through the process of delivering services to others. The results also suggested that the challenge of organizing peer support services could help prepare the peer service providers for future employment, both technically (e.g., organization ability and work habit) and mentally (e.g., dealing with stress).

The reported areas of improvement of peer service participants from their caregivers’ perspectives included: social communication skills, confidence in recovery, and illness stability-i.e., results consistent with the self-reported assessments of peer service providers and consumers. In addition, as the participants became more and more independent, the findings also indicated that peer service further benefited caregivers themselves with improved mood, reduced caring burden, and more confident in their family member’s recovery progress.

Participants from the later communities rated relatively higher satisfaction and perceived more benefits than those from the initial ones. This may reflect our being able to draw upon experiences gained from the initial communities. When developed peer support services for the initial communities, we also encountered the most common challenges faced in Western culture, including a lack of clear role definitions for peer service providers as well as for professionals such as community doctors [33]. We sought to allow peer service providers substantial latitude in their roles, as they reported feeling significant pressure when serving as a peer service provider for the first time. Thus, we did not limit the service schedule or the targeted content at first. We observed, however, that this apparent lack of structure did not decrease stress among peer service providers; indeed, it was our impression that it compounded whatever stress they experienced when fulfilling the supportive tasks associated with their consumers. The later assessed results of service satisfaction underscored our observation. Both peer service providers and consumers from the initial communities rated a relatively low score on richness of activity topics. As a result, when implementing peer support services in the later communities, we provided a schedule with a fixed service timetable, and offered peer service providers eight choices of service content. It is our sense that, in the later communities, the significantly higher satisfaction on service punctuality as well as richness of activity topic, and generally better feedback on all aspects of perceived service benefits, related directly to programming improvements derived from our initial experiences.

As for implications, this study revealed the outcomes of peer-delivered service under Chinese culture, which enriched its implementation and effectiveness under diverse contexts and customs. Furthermore, when evaluating service benefits, it targeted at a variety of key stakeholders including peer service providers, consumers, and their caregivers, which provided a more reliable and comprehensive understanding. Because the assessments were conducted at approximately 1 year after the service implementation, these results could support the sustainability and feasibility of peer service.

Some limitations in this study need to be addressed. First, our participation numbers in this study were small. While there were no apparent differences among those who dropped out and those who continued, it is uncertain how their views would have affected our apparent results. Second, although the measures used in this study were developed deliberately, most of them have not been validated. In addition, we used interval scales to query our participants; we realize that these do not offer definitive metrics to assure the equivalence of one interval, or one scale, with another. Nonetheless, the scales provided a structure to assist interviewing our participants and to compare the initial and later communities qualitatively. Third, due to difficulties with providing services to enough consumers, the study was conducted in two waves. Two initial communities were involved, and then another two were added. Although we compared the consistency and disparity between the two waves. Future studies should make more effort to reduce or control the potential biases introduced by unsimultaneous recruitment. Fourth, despite the study conducted follow-up assessment at 1 year after service implementation, effectiveness of service was only evaluated by retrospective interviews. Future studies should develop comparable measures and combine baseline and follow-up assessments to reveal the longitudinal effect. Fifth, even as we see positive outcomes in this small-scale demonstration, there will be a need for more research. Beijing, especially its resource-filled Chaoyang District, cannot be viewed as representative of all communities across China, especially those in rural regions. Future work must grapple with the extraordinary diversity of community resources, geography, transportation, and education-all key ingredients when offering care to persons with SMI. While a program such as ours may work well in an urban environment, it may be much more challenging in areas with a lower population density and convenient access to a central meeting site may be more difficult due to transportation or competing work needs. Finally, it will be essential to study in greater depth the roles of community doctors, who have relatively little training in mental health concerns. These clinicians are central to service delivery in China, and their observations were indispensable to charting the responses of peer service providers, consumers, and family members who often interact with them. Future research should must involve community doctors-both as sources of information and as potential participants themselves.

## Conclusions

Findings demonstrated that peer-delivered services could be effectively developed in multiple communities in China with high service satisfaction. The findings highlight that peer-delivered services have promise in China for benefiting persons with SMI and their family caregivers, as well as the peer service providers themselves. Further research is needed to assess the lasting impact of peer programs in China and to explore opportunities and barriers for broader implementation.

## Supplementary information

**Additional file 1: Table S1.** Descriptive statistics of the participants’ demographic at baseline for participants from initial and later communities. **Table S2.** Results of service satisfaction among peer service providers from initial and later communities at follow-up evaluation^a^. **Table S3.** Results of perceived benefit among peer service providers and consumers from initial and later communities at follow-up evaluation^a^. **Table S4.** Results of service satisfaction among consumers from initial and later communities at follow-up evaluation^a^.

## Data Availability

The dataset used and/or analyzed in this study is available from the corresponding author upon reasonable request.

## Abbreviation

SMI: Severe mental illness

## References

[1] Davidson L, Chinman M, Sells D, Rowe M (2006). Peer support among adults with serious mental illness: a report from the field. Schizophr Bull.

[2] Repper J, Carter T (2011). A review of the literature on peer support in mental health services. J Ment Health.

[3] Miyamoto Y, Sono T (2012). Lessons from peer support among individuals with mental health difficulties: a review of the literature. Clin Pract Epidemiol Ment Health.

[4] Trainor J, Shepherd M, Boydell KM, Leff A, Crawford E (1997). Beyond the service paradigm: the impact and implications of consumer/survivor initiatives. Psychiatr Rehabil J.

[5] Lawn S, Smith A, Hunter K (2008). Mental health peer support for hospital avoidance and early discharge: an Australian example of consumer driven and operated service. J Ment Health.

[6] Repper J, Aldridge B, Gilfoyle S, Gillard S, Perkins R, Rennison J (2013). Peer support workers: theory and practice: London: Centre for Mental Health.

[7] Ratzlaff S, McDiarmid D, Marty D, Rapp C (2006). The Kansas consumer as provider program: measuring the effects of a supported education initiative. Psychiatr Rehabil J.

[8] Weissman EM, Covell NH, Kushner M, Irwin J, Essock SM (2005). Implementing peer-assisted case management to help homeless veterans with mental illness transition to independent housing. Community Ment Health J.

[9] Yanos PT, Primavera LH, Knight EL (2001). Consumer-run service participation, recovery of social functioning, and the mediating role of psychological factors. Psychiatr Serv.

[10] Fukui S, Davidson LJ, Holter MC, Rapp CA (2010). Pathways to recovery (PTR): impact of peer-led group participation on mental health recovery outcomes. Psychiatr Rehabil J.

[11] Mahlke CI, Priebe S, Heumann K, Daubmann A, Wegscheider K, Bock T (2017). Effectiveness of one-to-one peer support for patients with severe mental illness - a randomised controlled trial. Eur Psychiatry.

[12] Magliano L, Fadden G, Madianos M, de Almeida JM, Held T, Guarneri M, Marasco C, Tosini P, Maj M (1998). Burden on the families of patients with schizophrenia: results of the BIOMED I study. Soc Psychiatry Psychiatr Epidemiol.

[13] Yesufu-Udechuku A, Harrison B, Mayo-Wilson E, Young N, Woodhams P, Shiers D, Kuipers E, Kendall T (2015). Interventions to improve the experience of caring for people with severe mental illness: systematic review and meta-analysis. Br J Psychiatry.

[14] Barker ET, Greenberg JS, Seltzer MM, Almeida DM (2012). Daily stress and cortisol patterns in parents of adult children with a serious mental illness. Health Psychol.

[15] Awad AG, Voruganti LN (2008). The burden of schizophrenia on caregivers: a review. Pharmacoeconomics.

[16] Solomon P (2004). Peer support/peer provided services underlying processes, benefits, and critical ingredients. Psychiatr Rehabil J.

[17] Tse S, Mak WWS, Lo IWK, Liu LL, Yuen WWY, Yau S, Ho K, Chan SK, Wong S (2017). A one-year longitudinal qualitative study of peer support services in a non-Western context: the perspectives of peer support workers, service users, and co-workers. Psychiatry Res.

[18] Lloyd-Evans B, Mayo-Wilson E, Harrison B, Istead H, Brown E, Pilling S, Johnson S, Kendall T (2014). A systematic review and meta-analysis of randomised controlled trials of peer support for people with severe mental illness. BMC Psychiatry.

[19] Fan Y, Ma N, Ma L, Xu W, Steven Lamberti J, Caine ED (2018). A community-based peer support service for persons with severe mental illness in China. BMC Psychiatry.

[20] National Bureau of Statistics N (2019). China statistics abstract in.

[21] Morosini P, Magliano L, Brambilla L, Ugolini S, Pioli R (2000). Development, reliability and acceptability of a new version of the DSM-IV social and occupational functioning assessment scale (SOFAS) to assess routine social funtioning. Acta Psychiatr Scand.

[22] Solomon P, Draine J (1995). One-year outcomes of a randomized trial of consumer case management. Eval Program Plann.

[23] Doherty I, Craig T, Attafua G, Boocock A, Jamieson-Craig R (2004). The consumer-employee as a member of a mental health assertive outreach team. II. Impressions of consumer-employees and other team members. J Ment Health.

[24] Kern RS, Zarate R, Glynn SM, Turner LR, Smith KM, Mitchell SS, Becker DR, Drake RE, Kopelowicz A, Tovey W (2013). A demonstration project involving peers as providers of evidence-based, supported employment services. Psychiatr Rehabil J.

[25] Mowbray CT, Moxley DP, Collins ME (1998). Consumers as mental health providers: first-person accounts of benefits and limitations. J Behav Health Serv Res.

[26] Sells D, Davidson L, Jewell C, Falzer P, Rowe M (2006). The treatment relationship in peer-based and regular case management for clients with severe mental illness. Psychiatr Serv.

[27] Davidson L, Bellamy C, Guy K, Miller R (2012). Peer support among persons with severe mental illnesses: a review of evidence and experience. World Psychiatry.

[28] Carpenter-Song E, Hipolito MMS, Whitley R (2012). **“**Right here is an oasis”: how “recovery communities” contribute to recovery for people with serious mental illnesses. Psychiatr Rehabil J.

[29] Kidd SA, Virdee G, Mihalakakos G, McKinney C, Feingold L, Collins A, Davidson L, Weingarten R, Maples N, Velligan D (2016). The welcome basket revisited: testing the feasibility of a brief peer support intervention to facilitate transition from hospital to community. Psychiatr Rehabil J.

[30] Cook JA, Copeland ME, Jonikas JA, Hamilton MM, Razzano LA, Grey DD, Floyd CB, Hudson WB, Macfarlane RT, Carter TM (2011). Results of a randomized controlled trial of mental illness self-management using wellness recovery action planning. Schizophr Bull.

[31] Rogers ES, Maru M, Johnson G, Cohee J, Hinkel J, Hashemi L (2016). A randomized trial of individual peer support for adults with psychiatric disabilities undergoing civil commitment. Psychiatr Rehabil J.

[32] Demissie M, Hanlon C, Birhane R, Ng L, Medhin G, Fekadu A (2018). Psychological interventions for bipolar disorder in low- and middle-income countries: systematic review. BJPsych Open.

[33] Kemp V, Henderson AR (2012). Challenges faced by mental health peer support workers: peer support from the peer supporter's point of view. Psychiatr Rehabil J.

