# Supplementary material for: Feasibility of peer support services among people with severe mental illness in China
Source: BMC Psychiatry. 2019 Nov 14;19:360. doi: 10.1186/s12888-019-2334-x (PMC6854628; doi:10.1186/s12888-019-2334-x)
Supplement: Supplementary file 1 — Additional file 1: Table S1. Descriptive statistics of the participants’ demographic at baseline for participants from initial and later communities. Table S2. Results of service satisfaction among peer service providers from initial and later communities at follow-up evaluationa. Table S3. Results of perceived benefit among peer service providers and consumers from initial and later communities at follow-up evaluationa. Table S4. Results of service satisfaction among consumers from initial and later communities at follow-up evaluationa. [file 12888_2019_2334_MOESM1_ESM.docx]

Table S1. Descriptive statistics of the participants’ demographic at baseline for participants from initial and later communities

| Demographic variables | Peer service provider | | Consumer | | |
| --- | --- | --- | --- | --- | --- |
|  | Initial communities (*n*=7) | Later communities (*n*=6) | | Initial communities (*n*=32) | Later communities (*n*=22) |
| Age | 39.43 (12.50) | 38.33 (5.79) | | 45.25 (8.97) | 48.55 (6.86) |
| Gender |  |  | |  |  |
| Female | 2 (29%) | 3 (50%) | | 22 (69%) | 9 (41%) |
| Male | 5 (71%) | 3 (50%) | | 10 (31%) | 13 (59%) |
| Education |  |  | |  |  |
| ≤ Middle school | 2 (29%) | 0 (0%) | | 9 (28%) | 4 (18%) |
| High school | 4 (57%) | 4 (67%) | | 14 (44%) | 11 (50%) |
| Some college | 1 (14%) | 0 (0%) | | 5 (16%) | 3 (14%) |
| College or more | 0 (0%) | 2 (33%) | | 4 (13%) | 4 (18%) |
| Current marital status |  |  | |  |  |
| Never married | 5 (71%) | 3 (50%) | | 12 (38%) | 16 (73%) |
| Currently married | 2 (29%) | 2 (33%) | | 11 (34%) | 3 (14%) |
| Divorced or separated | 0 (0%) | 1 (17%) | | 9 (28%) | 3 (14%) |
| Lives alone | 0 (0%) | 0 (0%) | | 7 (22%) | 3 (14%) |
| Current employment |  |  | |  |  |
| Yes | 2 (29%) | 2 (33%) | | 5 (16%) | 0 (0%) |
| No | 5 (71%) | 4 (67%) | | 27 (84%) | 22 (100%) |
| Diagnosis |  |  | |  |  |
| Schizophrenia | 5 (71%) | 3 (50%) | | 27 (84%) | 21 (96%) |
| Bipolar disorder | 2 (29%) | 3 (50%) | | 5 (16%) | 1 (5%) |
| First onset age | 18.86 (4.34) | 23.33 (5.75) | | 25.52 (8.55) | 24.64 (9.04) |

Table S2. Results of service satisfaction among peer service providers from initial and later communities at follow-up evaluation ^a^

| Service satisfaction | Initial communities (*n*=7) | Later communities (*n*=5) |
| --- | --- | --- |
| Overall work satisfaction |  |  |
| Overall, satisfied with the peer service | 7 (100%) | 5 (100%) |
| Time | 4.00 (0) | 4.20 (0.447) |
| Content | 3.00 (1.155) | 4.00 (0) |
| Environment | 4.14 (0.378) | 4.00 (0) |
| Relationship with consumers | 4.00 (0) | 4.20 (0.447) |
| Relationship with other peer service providers | 4.00 (0.577) | 4.40 (0.548) |
| Relationship with doctors involved | 5.00 (4.290) | 4.20 (0.447) |
| Work competency and stress |  |  |
| Qualified to be peer service providers | 5 (71%) | 5 (100%) |
| Feel stressful to be peer service providers | 3 (43%) | 3 (60%) |
| Continuous work willingness |  |  |
| Willing to continue | 6 (86%) | 5 (100%) |
| Confidence | 6.64 (2.734) | 7.80 (1.789) |

^a^ Category variables were tested by Chi-square (*χ^2^*) test, and continuous variables were tested by Mann Whitney U test.

Table S3. Results of perceived benefit among peer service providers and consumers from initial and later communities at follow-up evaluation ^a^

|  | Peer service provider | | Consumer | |
| --- | --- | --- | --- | --- |
|  | Initial communities (*n*=7) | Later communities (*n*=5) | Initial communities (*n*=26) | Later communities (*n*=16) |
| Self-perceived benefit |  |  |  |  |
| Disease related |  |  |  |  |
| Know more disease knowledge | 2 (29%) | 4 (80%) | 4 (15%) | 9 (56%) |
| Disease become more stable | 0 (0%) | 3 (60%) | 2 (8%) | 11 (69%) * |
| Social communication |  |  |  |  |
| Improve social communication skill | 4 (57%) | 4 (80%) | 9 (35%) | 9 (56%) |
| Improve relationship with families | 0 (0%) | 3 (60%) | 0 (0%) | 9 (56%) * |
| Care and support more for others | 1 (14%) | 4 (80%) | 2 (8%) | 9 (56%) * |
| Ability of daily life and work |  |  |  |  |
| Improve self-living ability | 0 (0%) | 4 (80%) | 2 (8%) | 8 (50%) * |
| Improve work skill | 7 (100%) | 3 (60%) | 4 (15%) | 5 (31%) |
| Emotion and self-perception |  |  |  |  |
| More confidence about recovery | 1 (14%) | 5 (100%) | 1 (4%) | 10 (63%) * |
| Improve mood and feeling cared about | 2 (29%) | 5 (100%) | 10 (39%) | 13 (81%) |
| Improve sense of belonging | 0 (0%) | 4 (80%) | 5 (19%) | 8 (50%) |
| Perceived benefit from others perspective |  |  |  |  |
| Family members | 5 (71%) | 4 (80%) | 5 (19%) | 14 (88%) * |
| Friends | 0 (0%) | 2 (40%) | 2 (8%) | 8 (50%) * |
| Community doctors | 3 (43%) | 4 (80%) | 2 (8%) | 14 (88%) * |
| Other community staff | 1 (14%) | 3 (60%) | 2 (8%) | 11 (69%) * |

a The numbers showed in this table represent how many participants answered “yes” on each particular question.

Asterisk (*) indicates significant difference between the initial communities and the later communities at the Bonferroni-corrected threshold of *p* value < 3.57 × 10^-3^.

Table S4. Results of service satisfaction among consumers from initial and later communities at follow-up evaluation ^a^

| Service satisfaction | Initial communities (*n*=26) | Later communities (*n*=16) |
| --- | --- | --- |
| Overall work satisfaction |  |  |
| Overall, satisfied with the peer service providers and service | 23 (89%) | 16 (100%) |
| Peer service providers’ competence | 3.68 (0.900) | 4.00 (0.632) |
| Peer service providers’ speak manners | 3.88 (0.440) | 4.13 (0.619) |
| Peer service providers’ disease stability | 3.72 (0.542) | 4.06 (0.574) |
| Service punctuality | 3.84 (0.374) | 4.31 (0.479) * |
| Richness of activity topic | 3.40 (0.913) | 4.06 (0.574) * |
| Continuous participation willingness | 21 (81%) | 15 (94%) |

^a^ Category variables were tested by Chi-square (*χ^2^*) test, and continuous variables were tested by *t* test.

Asterisk (*) indicates significant difference between the initial communities and the later communities at the Bonferroni-corrected threshold of *p* value < 0.01.
